# Methylation analysis of *DCC* gene in saliva samples is an efficient method for non-invasive detection of superficial hypopharyngeal cancer

**DOI:** 10.1038/s41416-024-02654-2

**Published:** 2024-03-27

**Authors:** Ryosuke Hirai, Hideaki Kinugasa, Shumpei Yamamoto, Soichiro Ako, Koichiro Tsutsumi, Makoto Abe, Koji Miyahara, Masahiro Nakagawa, Motoyuki Otsuka

**Affiliations:** 1 Department of Gastroenterology and Hepatology, Okayama University Graduate School of Medicine, Dentistry and Pharmaceutical Sciences, 2-5-1, Shikata, Kitaku, Okayama, Okayama, 700-8558 Japan; 2 Department of Internal Medicine, Hiroshima City Hospital, 7-33, Motomachi, Nakaku, Hiroshima, Hiroshima, 730-8518 Japan

**Keywords:** DNA methylation, Oral cancer detection, Cancer screening

## Abstract

**Background:**

Advances in upper gastrointestinal endoscopic technology have enabled early detection and treatment of hypopharyngeal cancer. However, in-depth pharyngeal observations require sedation and are invasive. It is important to establish a minimally invasive and simple evaluation method to identify high-risk patients.

**Methods:**

Eighty-seven patients with superficial hypopharyngeal cancer and 51 healthy controls were recruited. We assessed the methylation status of *DCC*, *PTGDR1*, *EDNRB*, and *ECAD*, in tissue and saliva samples and verified the diagnostic accuracy by methylation analyses of their promoter regions using quantitative methylation-specific PCR.

**Results:**

Significant differences between cancer and their surrounding non-cancerous tissues were observed in the methylation values of *DCC* (*p* = 0.003), *EDNRB* (*p* = 0.001), and *ECAD* (*p* = 0.043). Using receiver operating characteristic analyses of the methylation values in saliva samples, *DCC* showed the highest area under the curve values for the detection of superficial hypopharyngeal cancer (0.917, 95% confidence interval = 0.864–0.970), compared with those for *EDNRB* (0.680) and *ECAD* (0.639). When the cutoff for the methylation values of *DCC* was set at ≥0.163, the sensitivity to detect hypopharyngeal cancer was 82.8% and the specificity was 90.2%.

**Conclusions:**

*DCC* methylation in saliva samples could be a non-invasive and efficient tool for early detection of hypopharyngeal cancer in high-risk patients.

## Introduction

Head and neck squamous cell carcinoma (HNSCC) is the sixth most prevalent cancer worldwide, with 878,000 new cases and 364,000 deaths in 2020 [1]. HNSCC is usually diagnosed at an advanced stage, and the five-year survival rate of approximately 40% [2–4]. In particular, hypopharyngeal cancer is the most aggressive, lethal, and has the worst prognosis among HNSCC patients [5]. The anatomic proximity of the larynx, diagnosis at advanced stages, and higher rates of regional and distant metastases portend a worse prognosis than other HNSCC [6]. Advanced HNSCC is often treated with laryngopharyngeal esophagectomy and chemoradiotherapy; nevertheless, these treatments may greatly impair patients’ quality of life, causing both cosmetic and functional loss [7, 8]. Therefore, early detection and treatment are essential for HNSCC, especially hypopharyngeal cancer.

With the progress in upper gastrointestinal endoscopic technology, gastroenterologists have reported the importance of early endoscopic diagnosis and treatment of hypopharyngeal cancer [9–11]. Endoscopic submucosal resection (ESD) and endoscopic laryngopharyngeal surgery (ELPS) are widely accepted as safe and effective treatments for HNSCC, especially hypopharyngeal cancer [12, 13]. Conversely, endoscopy under sedation and analgesia is preferred because endoscopic pharyngeal observation is accompanied by pain, such as the gag reflex [14]. It is not feasible to perform detailed pharyngeal observations without sedation in patients undergoing an upper gastrointestinal endoscopy. In addition, it is not practical to use sedation for all patients owing to cost, space, and safety concerns. Therefore, there is an urgent need to establish a minimally invasive and straightforward evaluation method to screen high-risk patients with hypopharyngeal cancer.

DNA promoter hypermethylation, characterized by the reversible addition of a methyl group to the carbon-5 position of cytosine in cytosine-phosphate-guanine dinucleotides [15], is one of the major mechanisms involved in the transcriptional inactivation of certain carcinoma-associated genes, including HNSCC [16–18]. Several studies have attempted to detect HNSCC by analyzing DNA methylation in tissue and saliva [19–21]. However, these studies mainly targeted advanced cancers that require highly invasive treatment, and non-invasive screening methods have not been established for superficial cancers that can be locally resected. Ideally, cancer should be diagnosed accurately, particularly during the early asymptomatic stages. However, this has not been possible thus far because of difficulties in identifying such rare cases. If it was possible to diagnose superficial cancer by evaluating aberrant DNA methylation in saliva samples, this would be a breakthrough diagnostic tool in terms of being minimally invasive and easy to collect.

In this study, in collaboration with the departments of Gastroenterology, Otorhinolaryngology, and Head and Neck Surgery, we examined the feasibility of detecting hypopharyngeal superficial cancer by analyzing methylation of DNA promoter regions in saliva samples.

## Materials and methods

### Study design

Between January 2019 and March 2023, 87 patients with superficial hypopharyngeal cancer who underwent pharyngeal ESD or ELPS at Okayama University Hospital (61 cases) and Hiroshima City Hospital (26 cases), Japan, were recruited. In this study, superficial cancer was defined as cancer in situ or cancer with invasion of the subepithelial layer (not the muscular layer) [22].

First, DNA was extracted from the superficial hypopharyngeal cancer area and the surrounding normal mucosal area in tissue samples resected at Okayama University Hospital. The accuracy of the discrimination between cancerous and normal mucosa was examined using DNA promoter methylation. Second, in a derivation study, DNA methylation values were analyzed using the salivary DNA of 61 patients before endoscopic treatment (derivation cohort) and 51 healthy control subjects (control cohort) at Okayama University Hospital. After establishing the cutoff value for differentiating between patients and healthy subjects, the diagnostic accuracy was evaluated. Third, as a validation study, we analyzed DNA methylation levels in the salivary DNA of 26 patients who underwent endoscopic treatment for superficial hypopharyngeal cancer at Hiroshima City Hospital. We determined the detection rate in patients using the cutoff value calculated in the derivation study.

Demographic information of the patients and healthy control subjects was collected, including sex, age, and risk factors for malignancy, such as tobacco use, alcohol consumption, and body mass index (BMI). This study was conducted in accordance with the guidelines of the Declaration of Helsinki. All the patients provided written informed consent for the recommended procedures. The Okayama University Ethics Committee approved the study protocol (approval number:2006-001).

### Sample collection and DNA extraction

Saliva samples were collected from all 87 patients before endoscopic treatment and 51 healthy controls. Sample collection and DNA extraction were performed using an Oragene DISCOVER DNA Collection Kit (DNA Genotek Inc., Canada) according to the manufacturer’s instructions. Two milliliters of saliva were collected from each subject before breakfast and stored at room temperature after DNA extraction. All DNA was eluted in a final volume of 30 μl and stored at –30 °C. The DNA concentration and quality were assessed using a NanoDrop 2000 (Thermo Fisher Scientific, Waltham, USA).

Genomic DNA was extracted from seven slices of 10 μm thick sections of formalin-fixed paraffin-embedded (FFPE) tissues of resected superficial hypopharyngeal cancer using the QIAamp DNA FFPE Kit (Qiagen, Valencia, CA, USA), according to the manufacturer’s instructions. In 45 of the 61 patients in the derivation cohort, DNA extraction was performed separately for cancerous and non-cancerous areas of the resected samples. If the cancerous area spanned multiple FFPE sections, all sections were sliced and the DNA was homogenized. All DNA was eluted in a final volume of 30 μl and stored at –30 °C. The DNA concentration and quality were assessed using a NanoDrop 2000 (Thermo Fisher Scientific).

### Candidate gene selection

As candidate genes for the detection of hypopharyngeal cancer, we selected genes that were previously reported by comprehensive analyses [23, 24] and systematic reviews [25, 26] as being hypermethylated in tissue or saliva samples in advanced HNSCC cases. Four genes, Deleted in Colorectal Cancer (*DCC*) [21, 27], Prostaglandin D2 Receptor 1 (*PTGDR1*) [28, 29], Endothelin Receptor β (*EDNRB*) [21, 30], and E Cadherin (*ECAD*) [31, 32], were selected for this study. Primers were designed to specifically amplify the bisulfite-converted DNA of each gene. The forward and reverse sequences of each primer and β-actin (*ACTB*), as an internal reference gene, are listed in Supplementary Table S1. These primers were previously determined to detect the methylation status of target gene promoters [21, 28–32].

### Bisulfite modification and quantitative methylation-specific PCR (Q-MSP)

Sodium bisulfite conversion of DNA extracted from saliva and FFPE samples was conducted using an EZ DNA Methylation-Lightning Kit (Zymo Research, Irvine, CA, USA) according to the manufacturer’s protocol. For bisulfite conversion, 500 and 200 ng of DNA isolated from saliva and FFPE, respectively, were utilized. Bisulfite-converted DNA was stored at −80 °C.

Aberrant DNA methylation, which often occurs around the transcription start site (TSS) within a CpG island, was evaluated using Q-MSP with bisulfite-converted DNA as the template. The exon structure and CpG sites within the expanded views of the promoter region relative to the TSS are presented in Supplementary Fig. S1. The bisulfite-converted DNA was amplified and detected using a Roche LightCycler 96 system (Roche, Basel, Switzerland) under the following conditions: 95 °C for 10 min, followed by 45 cycles of 95 °C for 15 s and 60 °C for 1 min. A standard curve for Q-MSP was constructed by plotting known concentrations of serially diluted methylated and unmethylated standard DNA (EpiScope HCT116 g DNA; TaKaRa Bio, Kyoto, Japan). The normalized methylation value (NMV), representing the relative levels of methylation in each sample, was defined according to previous reports [28, 33, 34]: NMV = (each DNA-sample/each DNA-control)/(*ACTB*-sample/*ACTB*-control), where the DNA sample and DNA control represent target gene methylation levels in the tumor sample and in the universally methylated DNA control, respectively. *ACTB*-sample and *ACTB*-control correspond to *ACTB* in the sample and universally methylated DNA, respectively. The average of duplicate samples was used for analysis.

### Immunohistochemistry

Immunohistochemistry was performed using 4-μm-thick FFPE tissue sections from the derivation cohort and primary monoclonal antibodies for p16 (E6H4, prediluted; CIN Histology Kit, Roche, Heidelberg, Germany) as a surrogate marker for human papilloma viruses, and DCC (G97-449, dilution 100; BD Pharmingen, Franklin Lakes, NJ, USA). All stages of immunohistochemical staining were performed automatically using a BOND-MAX Automated Immunohistochemistry Vision Biosystem (Leica Microsystems GmbH, Wetzlar, Germany). Tissues were deparaffinized and pre-treated with the Epitope Retrieval Solution 2 (EDTA-buffer pH8.8) at 98 °C for 20 min. After washing steps, peroxidase blocking was carried out for 10 min using the Bond Polymer Refine Detection Kit DC9800 (Leica Microsystems GmbH). Tissues were again washed and then incubated with the primary antibody for 30 min. The immunohistochemical staining was observed by optical microscope and the p16 protein expression was considered positive (protein overexpression) when ≥70% of the tumor cells showed strong diffuse nuclear and cytoplasmic staining.

### Statistical analyses

The clinicopathological characteristics of the patients were compared using Fisher’s exact test. The proportions of DNA methylation in each gene were compared using the Student’s *t* test and Mann–Whitney U test. All tests were two-sided and considered statistically significant and clinically promising at *p* < 0.05. The cutoff value used to determine the presence of aberrant methylation was determined using receiver operating characteristic (ROC) analysis. The ROC curve was constructed by plotting sensitivity (true-positive rate) against 1—specificity (false-positive rate) for superficial hypopharyngeal cancer detection. The accuracy of sensitivity and specificity for each gene was evaluated using ROC analysis by calculating the area under the curve (AUC). Genes with AUC values > 0.65 were considered appropriate candidates for hypopharyngeal cancer detection. Logistic regression analysis was performed to calculate the odds ratio (OR) and 95% confidence interval (CI), and to evaluate factors associated with aberrant DNA methylation.

Statistical analyses were performed using EZR (Saitama Medical Center, Jichi Medical University, Saitama, Japan), which is a graphical user interface for R (R Foundation for Statistical Computing, Vienna, Austria).

## Results

### Patients’ characteristics

A total of 87 patients and 51 healthy controls were included in this study (Table 1). Almost all patients in the derivation cohort were males (93.4%), and their mean age was 67.4 years. Tobacco and alcohol consumption (current or past) were found in 80.3% and 91.8% of the patients, respectively. Tumor classification according to the WHO Classification of Tumors [35] was Tis to T1 in all but one case, and 56 (91.8%), 4 (6.6%), and 1 (1.6%) patients had pathological stages 0, I, and II, respectively. The p16 protein expression were positive in 4 (6.6%) patients. The control cohort, with no history of malignant disease, included 36 men (70.6%), 17 smokers (33.3%), and 26 drinkers (51.0%). There were significant differences between the derivation and control cohorts in terms of age, sex, smoking status, alcohol history, and BMI. In the comparison of patients’ characteristics between the derivation and validation cohorts, no significant difference was observed, except for BMI.

**Table 1:** Characteristics of the patients with hypopharyngeal superficial cancer and of the controls.

	Derivation cohort *n* = 61	Control cohort *n* = 51	*p* value*	Validation cohort *n* = 26	*p* value**
Age (years)	67.4 (±8.48)	54.5 (±17.1)	<0.01	67.0 (±10.6)	0.97
Gender (Men)	57 (93.4%)	36 (70.6%)	0.001	26 (100%)	0.31
Smoking status (Smoker^†^)	49 (80.3%)	17 (33.3%)	<0.01	24 (92.3)	0.21
Alcohol consumption (Drinker^††^)	56 (91.8%)	26 (51.0%)	<0.01	25 (96.2)	0.69
Body Mass Index	20 (14.4–28.7)	23.7 (18.3–28)	<0.01	21.9 (17.1–28.7)	0.012
Tumor diameter (mm)	17.7 (2–53)	–		19.3 (7–45)	0.54
Pathological T classification		–			
Tis	56 (91.8%)	–		23 (88.5%)	0.69
T1	4 (6.6%)	–		3 (11.5%)	
T2	1 (1.6%)	–		0	
T3/4	0	–		0	
Overall stage (UICC)		–			
0	56 (91.8%)	–		23 (88.5%)	0.69
I	4 (6.6%)	–		3 (11.5%)	
II	1 (1.6%)	–		0	
III/IV	0	–		0	
Esophageal cancer					
synchronous	7 (11.5%)	0		6 (23.1%)	0.059
past	47 (77.0%)	0		12 (46.2%)	
none	7 (11.5%)	51 (100%)		8 (30.7%)	
p16-IHC					
positive	4 (6.6%)	–		N/A	N/A
negative	57 (93.4%)	–		N/A	N/A

^*^Statistical significance between derivation cohort and control cohort.

**Statistical significance between derivation cohort and validation cohort.

†Smoker were defined as those who had ever smoked more than 100 cigarettes or had a continuous smoking history of at least six months.

††Drinker were defined as those who has ever consumed more than 20 g/day of alcohol for at least six months.

*IHC* immunohistochemical staining. *N/A* not available.

### Methylation values of the DNA promoter regions of the selected genes in FFPE tissue

**Fig. 1:**
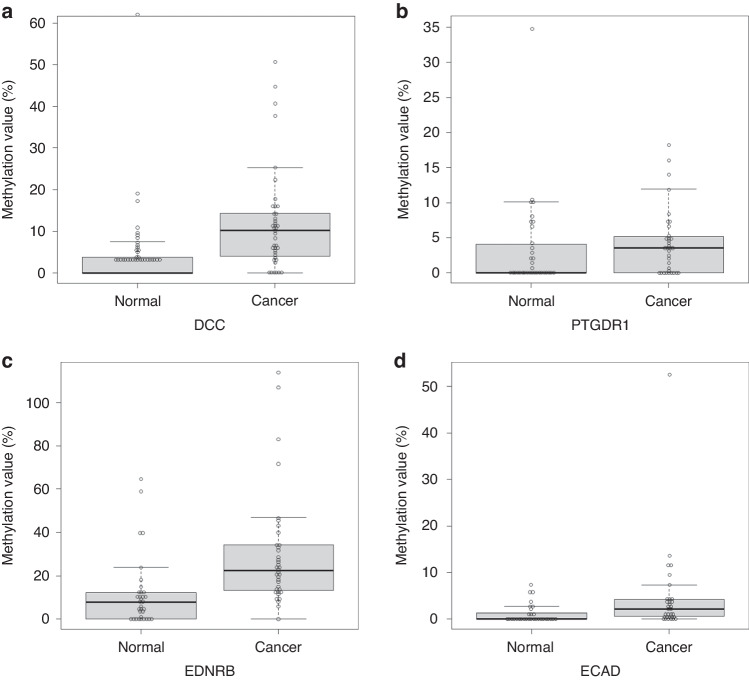
Methylation values of DNA in superficial hypopharyngeal cancerous tissues and in their surrounding non-cancerous tissues. Methylation values of genes in non-cancerous and cancerous tissues. **a** *DCC* (methylation value in non-cancerous tissues: 3.94, in cancer tissues: 12.1, *p* = 0.003), (**b**) *PTGDR1* (3.3, 4.33, *p* = 0.47), (**c**), *EDNRB* (11.84, 29.9, *p* = 0.001), (**d**), *ECAD* (1.09, 4.73, *p* = 0.043).

First, we analyzed the methylation status of the promoter regions of the selected genes in FFPE tissues of patients with hypopharyngeal cancer and in their surrounding normal mucosa in the derivation cohort. Among the four selected candidate genes, *DCC*, *PTGDR1*, *EDNRB*, and *ECAD*, the promoter regions of all genes except *PTGDR1* showed significantly higher methylation values in cancer areas than in normal mucosa areas (Fig. 1). We subsequently performed DNA methylation analyses using saliva samples for three genes (*DCC*, *EDNRB*, and *ECAD*).

### Methylation values in saliva samples from the derivation cohort

**Fig. 2:**
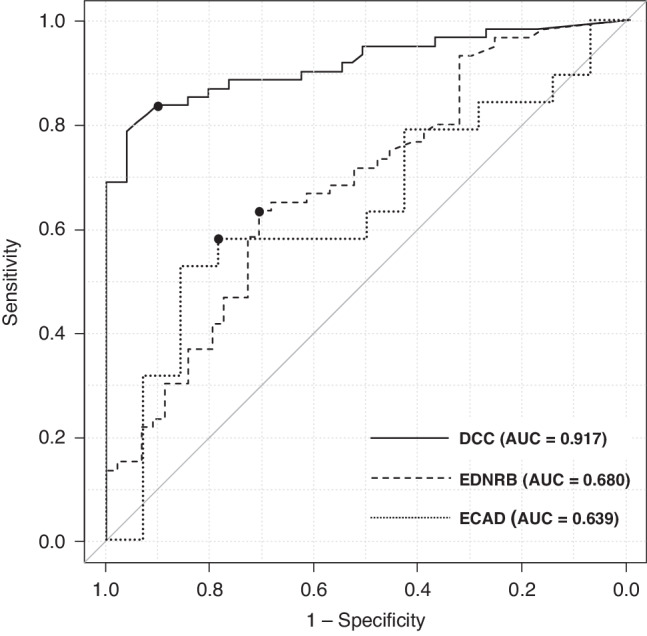
Comparison of the diagnostic accuracy for superficial hypopharyngeal cancer by the methylation analyses of the genes in the saliva. The black points in each chart indicate the position of the cutoff value. The AUC of the ROC analysis of *DCC*, *EDNRB*, and *ECAD* for superficial hypopharyngeal cancer diagnosis were 0.917 (95% CI:0.864–0.97), 0.680 (95% CI:0.576–0.784), and 0.639 (95% CI:0.441–0.838), respectively. The diagnostic accuracy of *DCC* methylation in saliva samples was significantly higher than that of *EDNRB* and *ECAD* methylation (p < 0.001, log-rank test). The cutoff values for the normalized methylation value (NMV) of *DCC*, *EDNRB*, and *ECAD* were ≥0.163, ≥0.256, and ≥0.655, respectively.

The diagnostic performances of *DCC*, *EDNRB*, and *ECAD* promoter methylation analyses in saliva samples to differentiate patients in the derivation cohort from those in the control cohort were compared using ROC analysis (Fig. 2). The AUCs of the ROCs curve analysis of *DCC* for the diagnosis of superficial hypopharyngeal cancer was 0.917 (95% CI:0.864–0.97). The AUC for the ROC of *EDNRB* and *ECAD* were 0.680 (95% CI:0.576–0.784) and 0.639 (95% CI:0.441–0.838), respectively. Among these three genes, NMVs were significantly higher in *DCC* (*p* < 0.001) and *EDNRB* (*p* = 0.007) in the derivation cohort than in the control cohort. There were no significant differences in the NMVs of *ECAD* (*p* = 0.234) between the two cohorts.

**Fig. 3:**
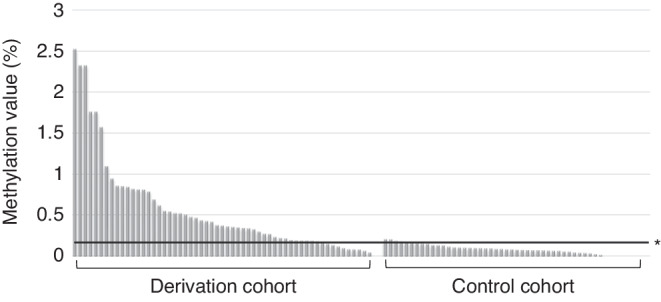
The methylation values of the *DCC* promoter region in the saliva samples from the derivation and control cohorts. The methylation values of the *DCC* promoter region in the salivary samples from the derivation cohort were significantly higher than those in the control cohort (*p* < 0.001). When the cut-off value of the *DCC* gene was set at NMV ≥ 0.163, the sensitivity, specificity, positive predictive value, negative predictive value, and accuracy to detect hypopharyngeal cancer were 83.6%, 90.2%, 91.1%, 82.1%, and 86.6%, respectively. * Cutoff value for detecting superficial hypopharyngeal cancer.

With the cutoff for the *DCC* methylation value in saliva samples set at *DCC* NMV $\ge$0.163, the sensitivity, specificity, positive predictive value (PPV), negative predictive value (NPV), and accuracy to detect hypopharyngeal cancer were 83.6%, 90.2%, 91.1%, 82.1%, and 86.6%, respectively (Fig. 3). The cutoff for *EDNRB* aberrant methylation in saliva samples was set at *EDNRB* NMV $\ge$0.256 and the sensitivity, specificity, PPV, NPV, and accuracy for *EDNRB* methylation analysis were 63.3%, 70.5%, 74.0%, 57.4%, and 65.4%, respectively.

Since the backgrounds of the patients in the derivation and control cohorts were significantly different, it is evident that factors such as lifestyle habits, age, and sex affect DNA promoter methylation. Thus, we evaluated the factors associated with increased methylation using multivariate logistic regression analyses (Table 2). Older age (OR, 2.53; 95% CI, 0.74–8.69), sex (1.89; 0.28–12.6), smoking history (0.65; 0.15–2.94), drinking history (2.38; 0.36–15.8), and obesity (0.37; 0.04–4.01) were not significant risk factors for *DCC* NMV $\ge$ 0.163. The presence of hypopharyngeal cancer was the only significant risk factor that exceeded the cutoff value (28.1; 6.47–122). Furthermore, when only the healthy controls registered in this study were examined, no significant differences were found in the values of salivary *DCC* methylation with respect to sex (*p* = 0.12), history of drinking (*p* = 0.22), smoking (*p* = 0.59), or BMI (*p* = 0.436) (Supplementary Fig. S2). A comparison of the *DCC* methylation values in cancer tissue and saliva samples from each case of the derivation cohort is shown in Supplementary Fig. S3.

**Table 2:** Multivariate analyses to detect risk factors for the increased *DCC* methylation above the cutoff value.

*DCC* NMV ≥ 0.163	OR (95% CI)	*p* value
Age ≥65	2.53 (0.74–8.69)	0.14
Sex (Male)	1.89 (0.28–12.6)	0.511
Smoking history	0.65 (0.15–2.94)	0.581
Drinking history	2.38 (0.36–15.8)	0.368
Obesity (BMI ≥25)	0.37 (0.04–4.01)	0.419
Hypopharyngeal cancer	28.1 (6.47–122)	0.00000851

### Validation study using saliva samples

**Fig. 4:**
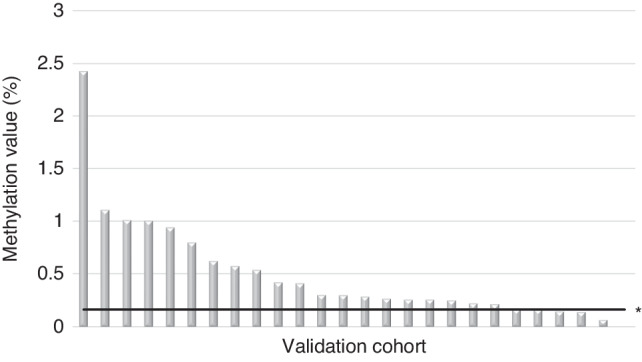
Validation study using the methylation values of the *DCC* promoter for the detection of superficial hypopharyngeal cancer in saliva samples. Methylation values of the *DCC* promoter region in salivary samples from the validation cohort. When the cutoff of the *DCC* gene promoter region was set at NMV ≥ 0.163, 22 of the 26 cases (84.6%) were detectable. * Cutoff value for detecting superficial hypopharyngeal cancer.

Using the cutoff values for the methylation status of *DCC* and *ENBDR* in saliva samples in the derivation study, we verified the diagnostic accuracy of the validation cohort for patients with superficial hypopharyngeal cancer. The mean NMV in the 26 cases of the validation cohort was 0.50 (0–2.43) for *DCC* and 0.52 (0.05–1.76) for *ENBDR*, respectively. Using a cut-off value of 0.163 for *DCC*, 22 cases (84.6%) in the validation cohort were detectable (Fig. 4), whereas 15 cases (57.7%) were detectable with a cut-off value of 0.256 for *EDNRB*.

### Methylation values of the DNA promoter region of the *DCC* gene in monitored saliva samples

In 12 patients with hypopharyngeal cancer, for which post-treatment saliva samples have been collected to date, we compared the methylation values of the *DCC* gene in saliva samples before and after cancer resection. The mean period from treatment to monitoring sampling was 20 (range, 2–32) months, and the mean post-treatment follow-up period was 39 (range, 10–50) months. The mean NMV of the *DCC* gene in the 12 patients before and after treatment was 0.450 (0.037–0.85) and 0.253 (0.06–0.61), respectively (Supplementary Fig. S4). Although the methylation values tended to decrease after treatment, there were no significant differences before and after treatment (*p* = 0.054). Even when patients were examined separately according to the presence or absence of recurrence (six patients each) during the observation period, neither group showed a significant decrease in the methylation value after treatment (*p* = 0.316 and 0.072, respectively). However, in recurrence-free cases, the post-treatment decline in methylation values was more pronounced than that in recurrent cases. Furthermore, it is noteworthy that in most cases, including those with no recurrence to date, the *DCC* methylation cutoff value set in this study was exceeded after treatment. This suggests that these cases could have the potential for metachronous recurrence, and long-term observation is required.

## Discussion

Despite the poor prognosis of hypopharyngeal cancer, there are no effective markers for its diagnosis or monitoring. In this study, methylation values of the DNA promoter region of the *DCC* gene in saliva samples showed high diagnostic accuracy for hypopharyngeal cancer, with a sensitivity of 83.6% and a specificity of 90.2%, even at the superficial cancer stage. This is a novel study that involves collaboration with multiple departments, such as gastroenterology, otolaryngology, and head and neck surgery, to verify epigenetic changes targeting superficial cancer, which has been difficult to evaluate owing to its rarity. This study will lead to the development of an extremely useful screening tool for head and neck cancer as an alternative to invasive endoscopic examinations.

*DCC* is a putative tumor suppressor gene located at 18q21 that encodes a transmembrane protein involved in both epithelial and neuronal cell differentiation [36]. Reestablishment of *DCC* expression has been shown to suppress tumorigenicity [37, 38], and hypermethylation of its promoter region has been detected not only in breast, gastric, and colon cancers, but also in oral squamous cell carcinoma [39–41]. Hypermethylation of the *DCC* promoter region of DNA in HNSCC is said to be correlated with *DCC* expression, and it has been previously shown that *DCC* expression is reduced or absent in tumors in which *DCC* were hypermethylated [39] (Supplementary Fig. S5). In this study, we determined that the DNA promoter region of *DCC* in saliva was hypermethylated in more than 80% of patients with superficial hypopharyngeal cancer and could be used as a reliable diagnostic marker. As saliva samples can be collected noninvasively and easily, screening methods utilizing these results may be advantageous as effective diagnostic tools.

In a previous study, hypermethylation of the promoter region of *EDNRB* was frequent in primary HNSCC and preferentially methylated in salivary rinses of patients with HNSCC (including 5% of hypopharyngeal cancers) [42]. Another study reported that, among 11 candidate genes, the promoter region of *ECAD* was highly methylated in both tissue and saliva samples of HNSCC patients (including 19% of hypopharyngeal cancer) [31]. Prostanoid receptors are involved in the development of many types of cancer, and hypermethylation of the promotor region of *PTGDR1* in tissue samples is highly correlated with recurrence in patients with hypopharyngeal cancer [28]. However, these studies have mainly targeted advanced cancers that require invasive treatment. In the present study, which uniquely focused on superficial hypopharyngeal cancer, the cancer diagnostic accuracy of the candidate genes described above was inferior to that of *DCC*. This result suggests that the genes targeted for DNA methylation evaluation might differ depending not only on their localization but also on their disease stage. In addition, although there were some cases in which saliva samples were hypermethylated despite low *DCC* methylation in carcinoma tissues and vice versa, *DCC* methylation in saliva was detectable in almost all cancer cases. To apply this technology in clinical practice in the future, further clarification of the factors influencing epigenetic changes in body fluids is needed.

The main limitation of this study was the significant difference in the background between the derivation and control cohorts. Nearly all patients with cancer were male, tended to have a lower BMI, and had a history of drinking and smoking. It has been reported that in addition to cancer, DNA methylation could be affected by lifestyle habits such as smoking and obesity [43]. However, multivariate logistic regression analysis showed that, the *DCC* methylation cut-off value set in this study was not significantly affected by patient background factors, including smoking and obesity. In addition, no significant differences in *DCC* promoter methylation due to sex or lifestyle habits were observed in healthy controls. Therefore, we conclude that aberrant methylation of *DCC* in the saliva of patients with superficial hypopharyngeal cancer is a reliable, disease-specific factor. Second, the selection of candidate genes for evaluation in this study was based on previous reports on the evaluation of methylation in advanced HNSCC. Therefore, additional useful genes may exist unanalyzed, and the best combination of these genes may not have been fully evaluated. The significance of this study is that it is the first to demonstrate the feasibility of screening for superficial hypopharyngeal cancer using methylation evaluation of salivary DNA. Based on the current results, we would like to further develop this study to establish optimal diagnostic markers by conducting a comprehensive analysis of DNA methylation and RNA sequencing. Third, although a downward trend in the *DCC* methylation value was confirmed after treatment, our study has not yet demonstrated its utility in disease monitoring using saliva samples. As only 12 post-treatment saliva samples have been collected to date, the present analysis was performed on these cases, and the sample size is undeniably small. However, the *DCC* methylation values after treatment were above the set cutoff in the majority of patients in both the non-recurrence and recurrence groups. This suggests that these patients could be at risk of recurrence during the long-term follow-up. We intended to continuously collect saliva samples from target cases and evaluate the feasibility of disease monitoring using saliva samples. Finally, both the derivation and validation cohorts were registered at a single center, and the number of cases was limited. It is necessary to accumulate more cases from multiple centers to verify the validity of these results.

In conclusion, DNA methylation analysis of *DCC* in patients with hypopharyngeal cancer from saliva samples showed high diagnostic accuracy, even at the superficial cancer stage. Because saliva can be collected easily and non-invasively, this method could be a useful tool for screening patients who are at high risk for hypopharyngeal cancer.

### Supplementary information

Supplemental Material

## Data Availability

All data presented in this work are present in the paper and/or in the Supplementary Materials. Further information and data of this study are available upon reasonable request from the corresponding author.
