# Supplementary material for: Methylation analysis of DCC gene in saliva samples is an efficient method for non-invasive detection of superficial hypopharyngeal cancer
Source: Br J Cancer. 2024 Mar 27;130(10):1725–31. doi: 10.1038/s41416-024-02654-2 (PMC11091138; doi:10.1038/s41416-024-02654-2)

Supplementary Table S1. Primer sequences used in this study for the methylation-specific PCR

Gene	Forward 5′-3′	Reverse 5′-3′	Amplicon size
DCC	CGCGATTTTTGGTTTCGAAGG	TACCGATTACTTAAAAATACGCG	128 bp
PTGDR1	TTTCGTACGTTATGAACTCGT	CGCCCATCACCGCCGAATTAC	81 bp
EDNRB	GGGAGTTGTAGTTTAGTTAGTTAGGGAGTAG	CCCGCGATTAAACTCGAAAA	75 bp
ECAD	TTAGGTTAGAGGGTTATCGCGT	TAACTAAAAATTCACCTACCGAC	173 bp
ACTB	TGGTGATGGAGGTTTAGTAAGT	AACCAATAAAACCTACTCCTCCCTTAA	133 bp

Supplementary Fig. S1 Schematic representation of *DCC*, *PTGDR1*, *EDNRB*, and *ECAD* genes

The exon structures and CpG sites were within the expanded views of the promoter region. Vertical lines, individual CpG sites; black box, relative location of primers used for Q-MSP; bent arrow, transcription start site (TSS); downward arrow, translation start site (ATG).

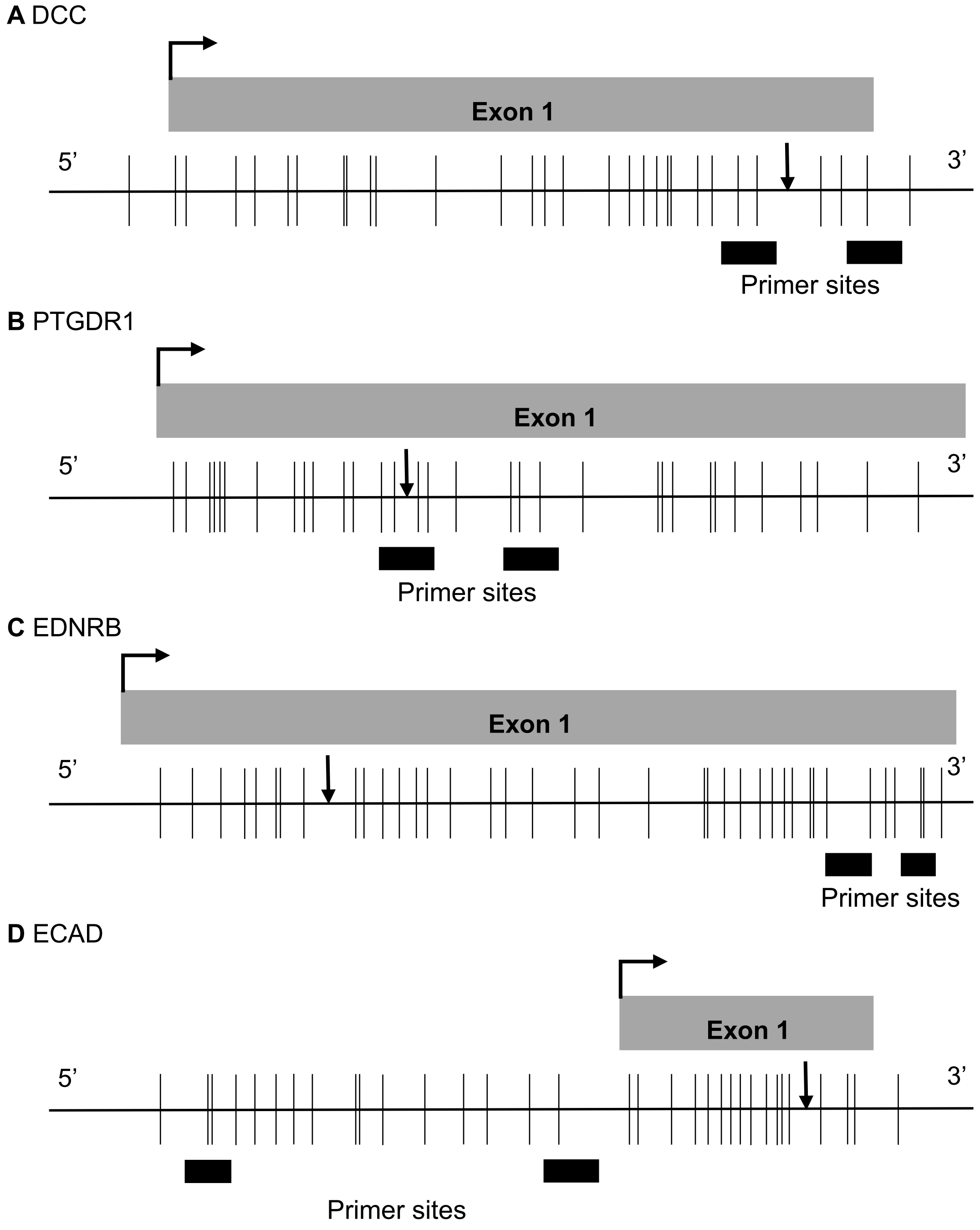

Supplementary Fig. S2 Differential methylation values of the *DCC* promoter region by the characteristics in the control cohort

No significant differences were observed in the mean methylation values of the *DCC* promoter region according to sex (A, methylation values in male:0.088, in female:0.061, p = 0.122), drinking history (B, drinker:0.090, non-drinker:0.070, p = 0.223), smoking history (C, smoker:0.086, non-smoker:0.077, p = 0.588), or BMI (D, obese:0.080, non-obese:0.080, p = 0.993).

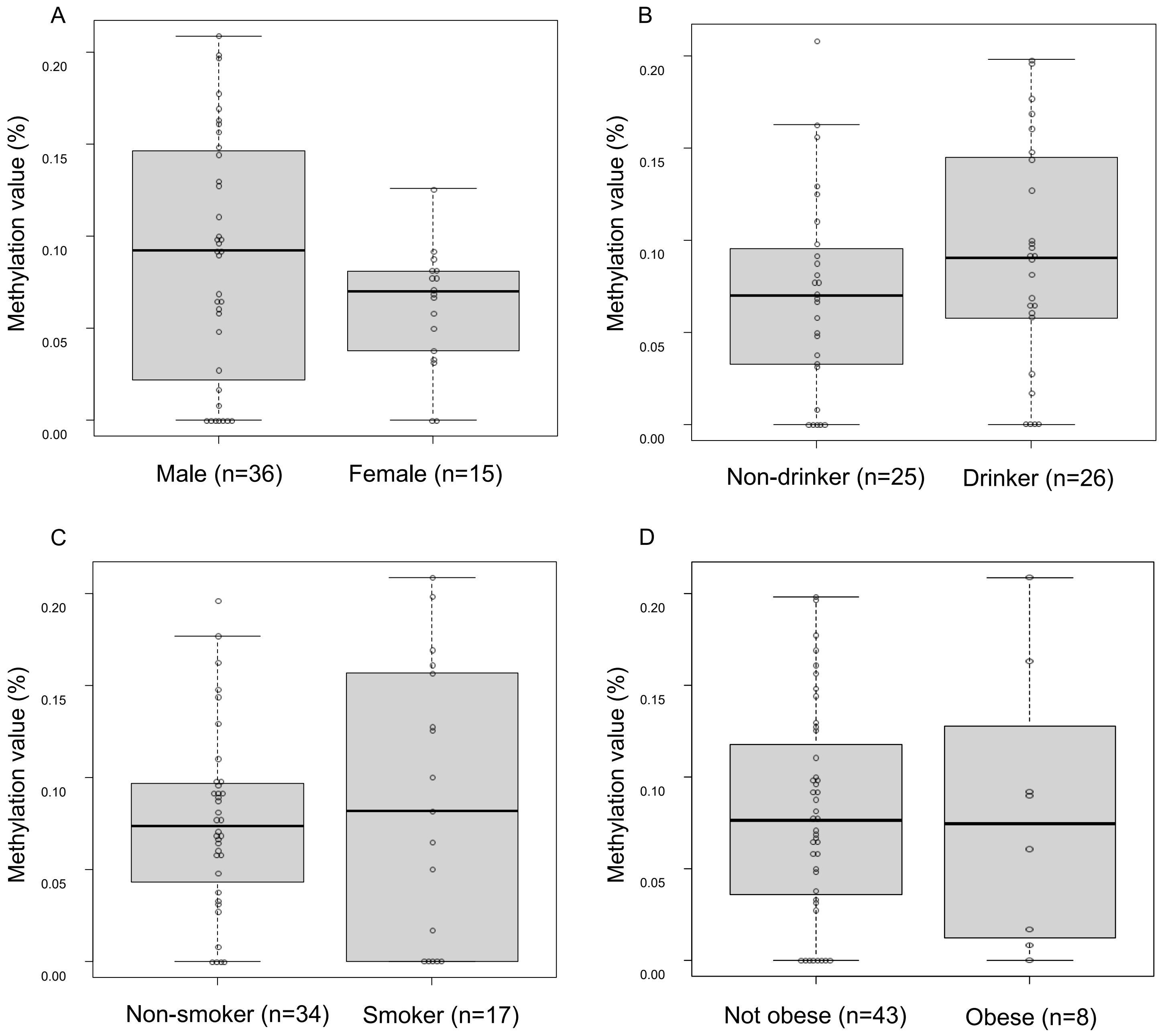

Supplementary Fig. S3 Comparison of the methylation values of the *DCC* promotor in tumor tissue and saliva samples in each case of derivation cohort

In each case, the thick gray line indicates the *DCC* methylation value in tumor tissue and the thin black line indicates the methylation value in saliva sample.

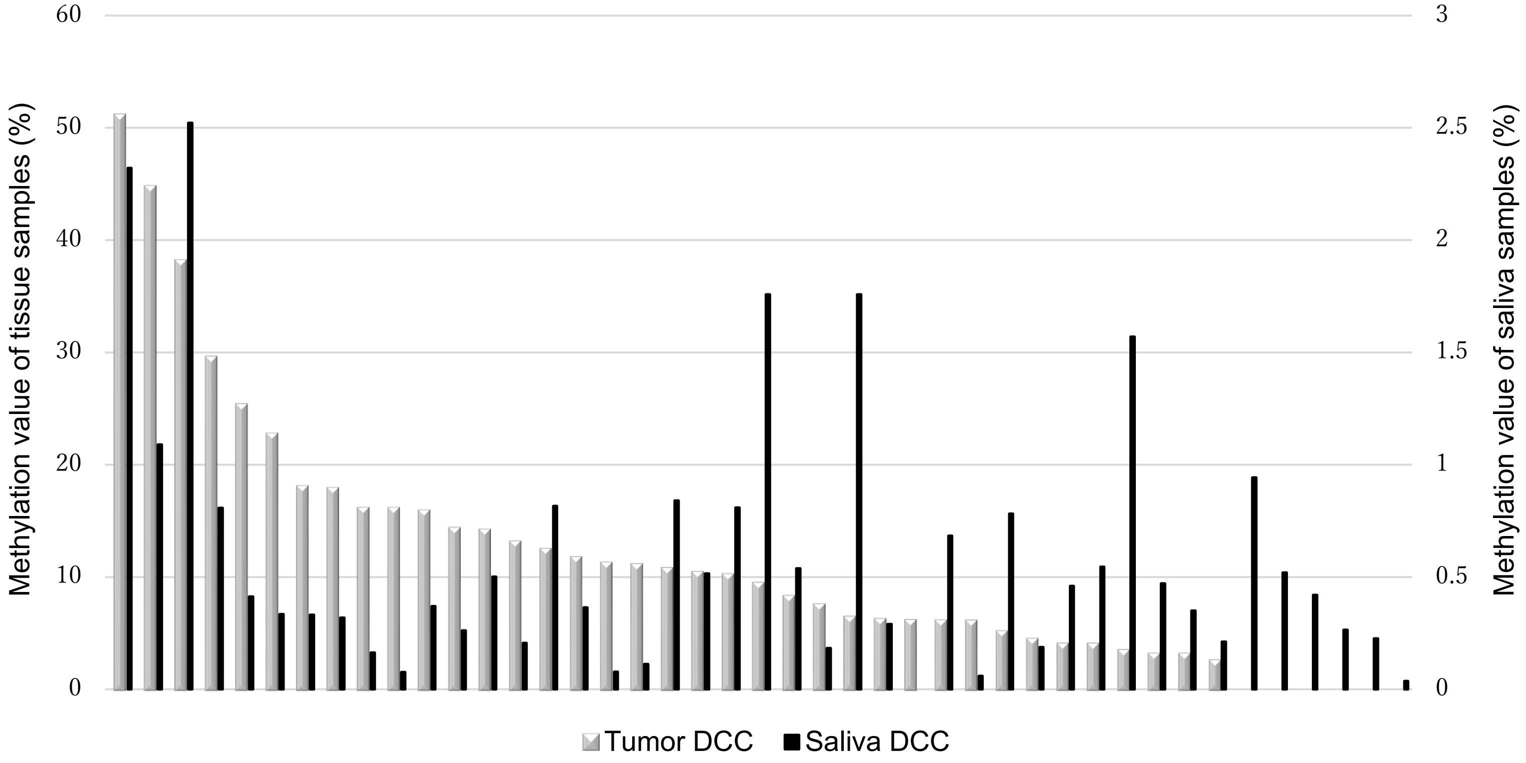

Supplementary Fig. S4 Changes in the methylation values of the DCC gene promoter in the salivary samples of 12 patients before and after treatment

Solid line: Saliva samples from six patients with no recurrence of hypopharyngeal cancer within the observation period (20–50 months). The mean DCC methylation values before and after treatment were 0.441 (0.163–0.854) and 0.229 (0.076–0.609), respectively; no significant differences were observed (p = 0.072). Dotted line: Saliva samples from six patients with hypopharyngeal cancer recurrence within the observation period (10–47 months). The mean DCC methylation values before and after treatment were 0.459 (0.037 –0.816) and 0.277 (0.06–0.609), respectively, and no significant differences were observed (p = 0.316).

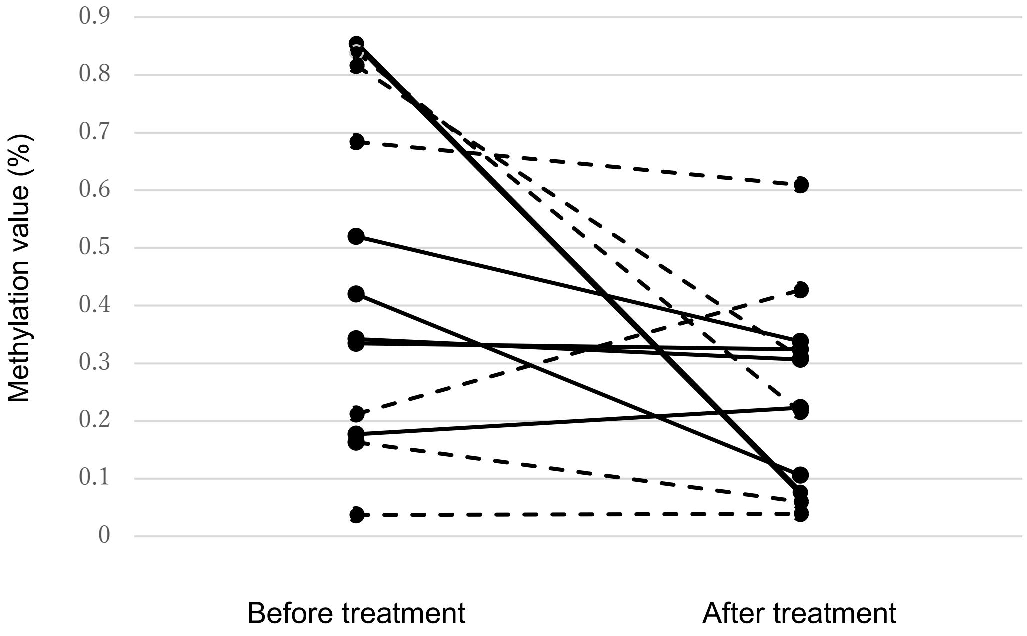

Supplementary Fig. S5 Representative photomicrograph of immunohistochemical staining for DCC in samples with and without *DCC* hypermethylation

A, Immunohistochemical staining image of a sample with no *DCC* methylation detected in the FFPE tissue. Diffuse staining was observed in the cytoplasm of tumour cells. B, Immunohistochemical staining image of a sample showing the highest methylation value of *DCC* in the FFPE tissue. Extensive staining defects were observed in the cytoplasm of tumour cells.

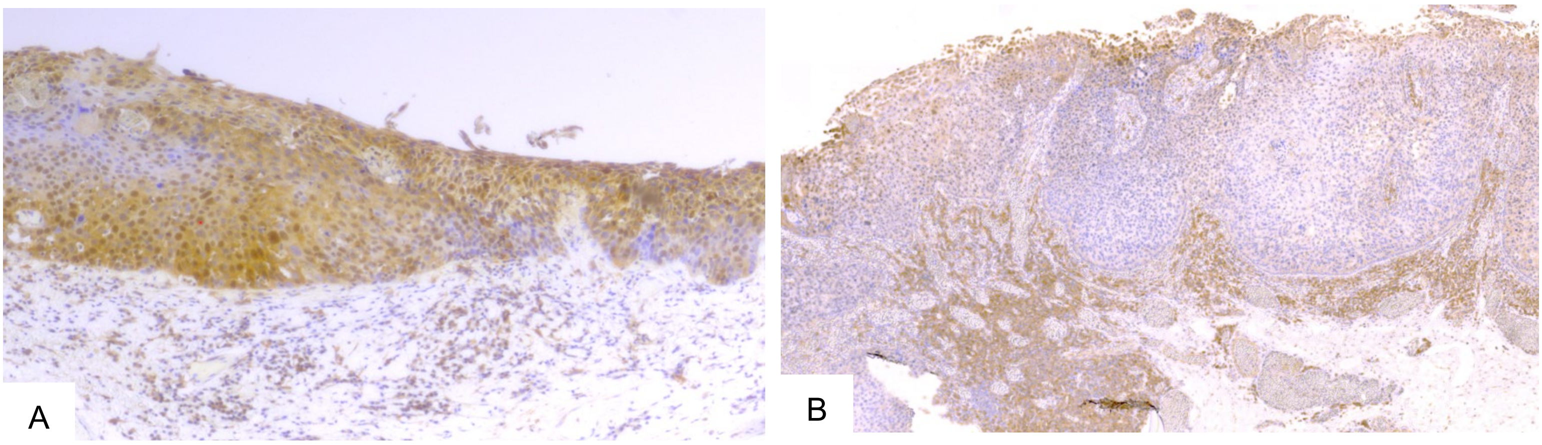

Supplement: Supplementary file 1 — Supplemental Material [file 41416_2024_2654_MOESM1_ESM.pdf]
